# Genome-Scale Metabolic Model of *Caldicellulosiruptor bescii* Reveals Optimal Metabolic Engineering Strategies for Bio-based Chemical Production

**DOI:** 10.1128/mSystems.01351-20

**Published:** 2021-06-01

**Authors:** Ke Zhang, Weishu Zhao, Dmitry A. Rodionov, Gabriel M. Rubinstein, Diep N. Nguyen, Tania N. N. Tanwee, James Crosby, Ryan G. Bing, Robert M. Kelly, Michael W. W. Adams, Ying Zhang

**Affiliations:** a Department of Cell and Molecular Biology, College of the Environment and Life Sciences, University of Rhode Island, Kingston, Rhode Island, USA; b Sanford-Burnham-Prebys Medical Discovery Institute, La Jolla, California, USA; c A.A. Kharkevich Institute for Information Transmission Problems, Russian Academy of Sciences, Moscow, Russia; d Department of Biochemistry and Molecular Biology, University of Georgia, Athens, Georgia, USA; e Department of Chemical and Biomolecular Engineering, North Carolina State University, Raleigh, North Carolina, USA; ExxonMobil Research and Engineering

**Keywords:** bio-based chemical production, *Caldicellulosiruptor*, central carbon metabolism, metabolic engineering, metabolic modeling, redox balance

## Abstract

Metabolic modeling was used to examine potential bottlenecks that could be encountered for metabolic engineering of the cellulolytic extreme thermophile Caldicellulosiruptor bescii to produce bio-based chemicals from plant biomass. The model utilizes subsystems-based genome annotation, targeted reconstruction of carbohydrate utilization pathways, and biochemical and physiological experimental validations. Specifically, carbohydrate transport and utilization pathways involving 160 genes and their corresponding functions were incorporated, representing the utilization of C5/C6 monosaccharides, disaccharides, and polysaccharides such as cellulose and xylan. To illustrate its utility, the model predicted that optimal production from biomass-based sugars of the model product, ethanol, was driven by ATP production, redox balancing, and proton translocation, mediated through the interplay of an ATP synthase, a membrane-bound hydrogenase, a bifurcating hydrogenase, and a bifurcating NAD- and NADP-dependent oxidoreductase. These mechanistic insights guided the design and optimization of new engineering strategies for product optimization, which were subsequently tested in the *C. bescii* model, showing a nearly 2-fold increase in ethanol yields. The *C. bescii* model provides a useful platform for investigating the potential redox controls that mediate the carbon and energy flows in metabolism and sets the stage for future design of engineering strategies aiming at optimizing the production of ethanol and other bio-based chemicals.

**IMPORTANCE** The extremely thermophilic cellulolytic bacterium, Caldicellulosiruptor bescii, degrades plant biomass at high temperatures without any pretreatments and can serve as a strategic platform for industrial applications. The metabolic engineering of *C. bescii*, however, faces potential bottlenecks in bio-based chemical productions. By simulating the optimal ethanol production, a complex interplay between redox balancing and the carbon and energy flow was revealed using a *C. bescii* genome-scale metabolic model. New engineering strategies were designed based on an improved mechanistic understanding of the *C. bescii* metabolism, and the new designs were modeled under different genetic backgrounds to identify optimal strategies. The *C. bescii* model provided useful insights into the metabolic controls of this organism thereby opening up prospects for optimizing production of a wide range of bio-based chemicals.

## INTRODUCTION

Global population growth and increasing industrial development have generated a greater demand for energy and resources (1). However, the supply of conventional fossil fuels is finite and their use is burdened with environmental issues. Therefore, their replacement with a sustainable energy supply becomes more and more pressing. Plant biomass can be used to generate fuels and other valuable chemicals (e.g., ethanol, biodiesel, plastics, and industrial chemicals) that can serve as renewable alternatives to many of the products derived from petroleum or natural gas and do so in environmentally benign processes. Inedible plants (such as switchgrass and poplar) are the ideal biomass sources for industrial biotechnology, since they are abundant, renewable, economical, and do not compete with food supply. However, plant biomass is extremely recalcitrant to deconstruction and thus requires chemical or enzymatic pretreatment to make the carbohydrate content accessible to prototypical industrial microbes. This creates an economic barrier for industrial development, as well as generating negative environmental ramifications (2). Clearly, alternative microbes that can degrade nonpretreated plant biomass, although rare in nature, are needed to meet this challenge through metabolic engineering to generate the needed biofuels and other bioproducts.

Caldicellulosiruptor bescii is a promising platform microorganism for converting lignocellulose to bio-based chemicals, based on its ability to degrade nonedible plant biomass, both cellulose and hemicellulose, without any enzymatic or chemical pretreatments (3, 4). *C. bescii* is an extremely thermophilic, strictly anaerobic, Gram-positive bacterium (5). It is the most thermophilic cellulolytic bacterium known to date with an optimal growth temperature (*T*_opt_) of 78 to 80°C and a maximum growth temperature (*T*_max_) of 90°C. *C. bescii* utilizes a wide range of simple and complex carbohydrates, both C5- and C6-based (5). Its high growth temperature creates several advantages for industrial applications, including (i) reduced risks of phage infection and contamination (since no known phages survive at such high temperatures), (ii) higher solubility of plant biomass polysaccharides, and (iii) the potential to distill volatile products directly from fermentation broths, which also minimizes product toxicity (6).

Engineered *C. bescii* strains are capable of producing ethanol (7–11) or acetone and H_2_ (12) by fermenting simple sugars (i.e., cellobiose and maltose), crystalline cellulose, or plant biomass (i.e., switchgrass and poplar) as the sole carbon and energy source. Wild-type *C. bescii* does not contain genes required for ethanol production, so foreign genes have been introduced through a uracil auxotrophy system enabled by development of *pyrFA* or *pyrE* mutants for the construction of engineered strains (13, 14). Recently, the *pyrE* deletion background, strain MACB1018, is shown to have a more stable genetic background than the *pyrFA* deletion background, strain JWCB005 (15). A lactate dehydrogenase (*ldh*) knockout strain, MACB1034, is constructed with the *pyrE* mutant, providing a stable genetic background, and is used as the parent strain for the ethanol production (14). A bifunctional acetaldehyde/alcohol dehydrogenase (AdhE) from Clostridium thermocellum (*T*_opt_ = 55 to 60°C) was expressed heterogeneously in *C. bescii*, enabling the engineered *C. bescii* strain to produce ethanol from crystalline cellulose, albeit at a suboptimal growth temperature of 60°C (10). In recent work, heterogeneous expression of genes encoding the enzymes, aldehyde ferredoxin oxidoreductase from Pyrococcus furiosus (*T*_opt_ = 100°C) and alcohol dehydrogenase (AdhA) from *Thermoanaerobacter* sp. strain X514 (*T*_opt_ = 72°C), enabled *C. bescii* to produce ethanol from crystalline cellulose optimally at 75°C (16). However, further development of *C. bescii* strains to produce bio-based chemicals at industrially relevant titers requires a better understanding of fermentation patterns to inform metabolic engineering.

Genome-scale models (GEMs) provide a systems-level view of the metabolism and are frequently applied to optimize the production of bio-based chemicals in engineered microorganisms. This approach has been used to model thermophilic bacteria that are capable of degrading cellulose or hemicellulose, such as Clostridium thermocellum (cellulose; *T*_opt_ = 55 to 60°C [17, 18]) and Thermoanaerobacterium saccharolyticum (hemicellulose; *T*_opt_ = 70°C [19]), as well as the mesophile Clostridium cellulolyticum (20), which degrades both cellulose and hemicellulose. In contrast to these organisms, *C. bescii* is much more thermophilic, growing up to 90°C (3) and, more importantly, utilizes C5-based hemicellulose, the second major carbohydrate in plant biomass, in addition to C6-based cellulose (21). This is a major advantage over thermophilic species that have only a cellulose-degrading or a hemicellulose-degrading metabolism (22–24). The adaptation of *C. bescii* to high temperature also represents an advantage over mesophilic species (i.e., Clostridium cellulolyticum) due to the better compatibility of thermophiles with industrial processing conditions.

Extensive biochemical and physiological studies of *C. bescii* have revealed several unique features in the central carbon and redox metabolism of this organism. Its central carbon metabolism is characterized by the presence of a glyceraldehyde-3-phosphate ferredoxin oxidoreductase (GOR), which uses ferredoxin as an electron carrier for oxidizing glyceraldehyde-3-phosphate (GAP) to 3-phosphoglycerate. The GOR pathway functions in parallel to the canonical GAP dehydrogenase (GAPDH), which uses NAD as the electron carrier (25). A second potentially confounding aspect to *C. bescii* metabolism is the fate of the reduced ferredoxin (Fd_red_) produced by GOR and pyruvate ferredoxin oxidoreductase (POR), since Fd_red_ can be oxidized by three redox related enzyme complexes (10, 26). One of these is conventional membrane-bound [NiFe]-hydrogenase (MBH), which couples Fd_red_ oxidation to H_2_ production (9, 27). However, the other two are examples of a recently discovered type of energy-conserving enzyme that carries out electron bifurcation wherein an exergonic reaction is used to drive an endergonic reaction (28). The first is a bifurcating [FeFe]-hydrogenase (BF-H_2_ase) that reversibly couples H_2_ production to energy-yielding Fd_red_ oxidation and energy-requiring NADH oxidation (29). The second example is NADH-dependent ferredoxin NADP oxidoreductase (BF-Nfn); this also simultaneously oxidizes Fd_red_ and NADH, while coupling these two reactions to the reduction of NADP for biosynthetic purposes (30). The complex interconversion of the oxidized and reduced forms of these electron carriers, and their respective connections to the central carbon metabolism of *C. bescii* suggests that these processes are tightly regulated (25). To enhance NADH generation in *C. bescii*, required for ethanol production using AdhE, we also expressed heterogeneously a membrane-bound, sodium ion-pumping reduced ferredoxin NAD oxidoreductase (Rnf_Na) from *Thermoanaerobacter* sp. strain X514 (*T*_opt_ = 60°C). It was hypothesized that this would use Fd_red_ produced from carbohydrate oxidation to increase the production of NADH for NADH-dependent ethanol production; however, the introduction of Rnf_Na had only a marginal effect in enhancing ethanol yields (10).

Further enhancement of ethanol production in *C. bescii* obviously requires a fundamental understanding of the redox processes at a systems level, and that is the goal of the research described here. Overall, the unique metabolic and physiological characteristics of *C. bescii* motivated the application of GEM reconstruction for guiding the metabolic engineering designs of this organism for bioproduct generation. This study represents the first metabolic modeling of *C. bescii* using a subsystems-level approach. Model validations were performed using experimental physiological and metabolic data. The *C. bescii* model was further refined to accurately simulate use of carbon and energy sources, including both simple (glucose, cellobiose) and complex (cellulose, xylan) carbohydrates. The model was then used to identify metabolic bottlenecks and predict novel engineering strategies for the optimization of bio-based chemicals by resolving constraints in redox balancing.

## RESULTS

### Overview of the genome-scale metabolic model GEM-iCbes.

The genome-scale metabolic model of *C. bescii*, GEM-iCbes, contains 610 genes, 718 metabolites, and 714 metabolic reactions (see Data Set S1 in the supplemental material). Compared to existing models of other plant biomass-degrading thermophilic bacteria that can use either cellulose or hemicellulose (but not both), the GEM-iCbes included a higher percentage of protein-coding genes (CDS) over the genome (24%) and across all metabolic genes predicted by the COG classification (87%) (Table 1). Reactions in the GEM-iCbes included 589 cytosolic reactions, 60 transmembrane reactions, and 12 extracellular reactions, all associated with specific gene annotations. A high consistency score (99%) was achieved with GEM-iCbes when analyzed using the Memote consistency check (31), which is comparable to the consistency score of 98%, for a recently updated model of *C. thermocellum*, iCBI655 (18) (Table 1).

**TABLE 1:** Comparison of the *C. bescii* model with existing models of plant-biomass degrading bacteria^*a*^

Parameter	Caldicellulosiruptor bescii DSM 6725	Clostridium thermocellum DSM 1313	Clostridium thermocellum ATCC 27405	Thermoanaerobacterium saccharolyticum JW/SL-YS485	Clostridium cellulolyticum H10
Physiology					
Growth temp, range (optimal) (°C)	42–90 (78)	40–60 (50–55)	50–68 (60)	45–70 (60)	25−45 (34)
Plant biomass	Cellulose and hemicellulose	Cellulose	Cellulose	Hemicellulose	Cellulose and hemicellulose
Genome					
Size (Mbp)	2.92	3.56	3.84	2.83	4.07
No. of CDS	2,565	2,939	3,218	2,407	3,334
GEM					
Model ID	GEM-iCbes	iCBI655	iCth446	T. saccharolyticum model	iFS431
No. of genes	610	665	446	315	431
% CDS in model	24	23	14	13	13
% COG metabolic genes in model	87	80	53	34	42
No. of genes of carbohydrate transport and utilization	160	100	55	22	69
No. of metabolites	718	795	599	503	603
No. of reactions (exclude exchange)	714	795	607	515	584
No. of gene-associated reactions	661	689	458	461	513
% gene-associated reactions	93	87	75	90	88
Consistency (%)	99	98	ND	85	57
Stoichiometry consistency (%)	100	100	ND	99	100
Mass balance (%)	99	97	ND	93	0
Charge balance (%)	99	97	ND	94	0
Metabolite connectivity (%)	100	100	ND	100	99
Bounded flux in default medium (%)	93	96	ND	11	1
Reference(s)	This study	18, 24	17, 61	19, 62	20, 62

a Growth temperature indicates both the growth range and the optimal temperatures (in parentheses). The number of CDS indicates the count of protein-coding sequences in the genome. The consistency scores were calculated using the Memote software (v0.11.1) for standardized model assessment (31). ND, no data were available due to incompatibility of the model with Memote.

10.1128/mSystems.01351-20.1 DATA SET S1 Detailed representation of the genome-scale metabolic model of *C. bescii*. Download Data Set S1, XLSX file, 0.3 MB. Copyright © 2021 Zhang et al. 2021 Zhang et al. https://creativecommons.org/licenses/by/4.0/ This content is distributed under the terms of the Creative Commons Attribution 4.0 International license.

The metabolic reconstruction of *C. bescii* involved extensive manual curation, combining evidence from putative gene orthology and biochemical/physiological experiments in *C. bescii* and other thermophilic or Gram-positive bacteria. The experimentally verified biochemical functions included the ferredoxin-dependent oxidoreductase GOR (25), a phosphoglycerate kinase (PGK) that uses either ADP or GDP as cofactors (see Data Set S2), a GTP-forming phosphoenolpyruvate carboxykinase (PCK) (see Data Set S2), as well as a unique combination of three redox-related enzymes that all use ferredoxin as an electron carrier: MBH, BF-H_2_ase, and BF-Nfn (9, 25, 32). In addition, the *C. bescii* model integrated utilization pathways of various simple and complex carbohydrates, including monosaccharides of both C5 (e.g., xylose and arabinose) and C6 (e.g., glucose, fructose, and galactose), disaccharides (e.g., lactose, maltose, and cellobiose), as well as a variety of plant polysaccharides, including both cellulose and hemicellulose (xylan). Functional roles of extracellular enzymes that contribute to polysaccharide degradation, including 15 glycosyl hydrolases (GHs) and three polysaccharide lyases (PLs), were manually curated and incorporated into the model. The extracellular GHs are responsible for the degradation of cellulose, xylan, mannans, and starch, while the PLs are required for pectin degradation. Uptake of oligo- and monosaccharides involves 18 ABC superfamily transporters encoded by 65 genes. The reconstructed carbohydrate catabolic pathways involve at least 77 presumably cytoplasmic enzymes, including 37 GHs. Specific transcriptional regulators controlling these carbohydrate utilization pathways were excluded from the model; their transcriptional regulons, including DNA-binding sites and carbohydrate utilization genes, and operons are presented in the accompanying paper (33). Compared to the existing models of related cellulose or hemicellulose-degrading species, the *C. bescii* model represented an expansion of 160 genes involved in the carbohydrate transport and catabolism (Table 1), the largest functional subsystem represented in the model (see Fig. S1).

10.1128/mSystems.01351-20.2 DATA SET S2 Experimental data and model configurations used for the validation of the *C. bescii* model. Download Data Set S2, XLSX file, 0.6 MB. Copyright © 2021 Zhang et al. 2021 Zhang et al. https://creativecommons.org/licenses/by/4.0/ This content is distributed under the terms of the Creative Commons Attribution 4.0 International license.

10.1128/mSystems.01351-20.5 FIG S1 Subsystems distribution of metabolic genes incorporated in the genome-scale model of *C. bescii*. Download FIG S1, TIF file, 0.5 MB. Copyright © 2021 Zhang et al. 2021 Zhang et al. https://creativecommons.org/licenses/by/4.0/ This content is distributed under the terms of the Creative Commons Attribution 4.0 International license.

### Model validation.

**FIG 1:**
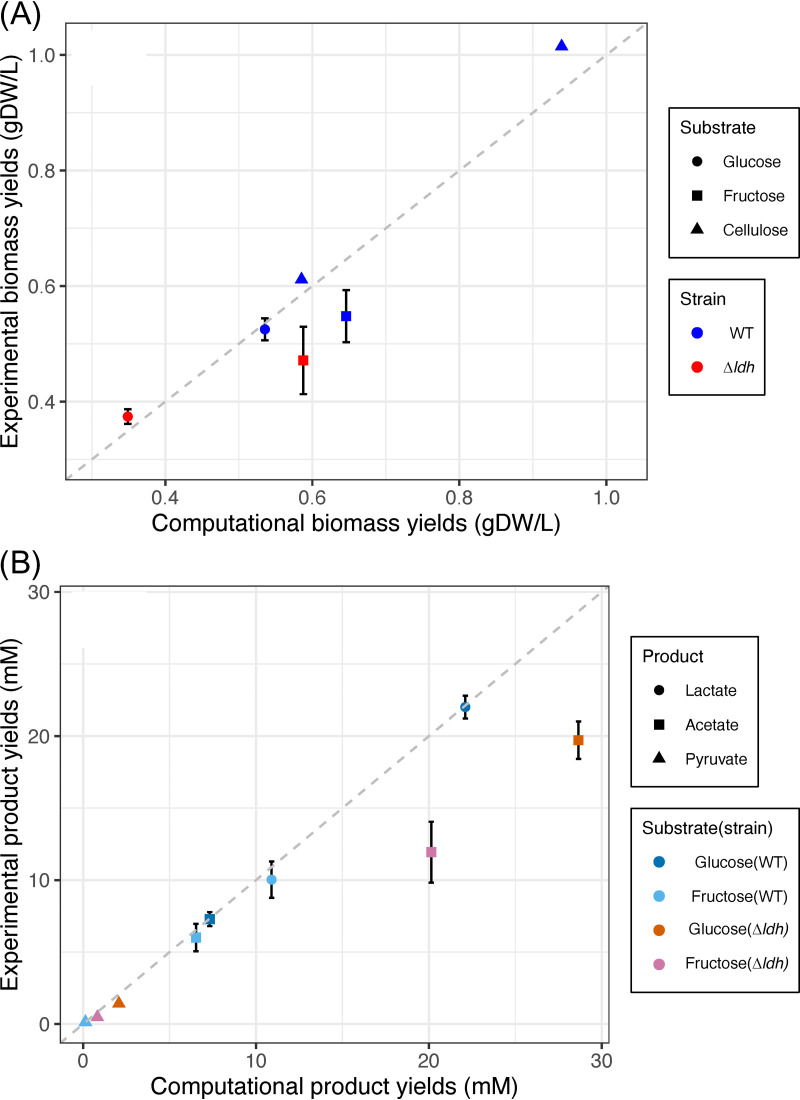
Experiment-based model validation using experimental measurements from WT and *Δldh* using glucose and fructose as sole carbon sources (5, 15). (A) Growth yield validation; (B) product yield validation. A dashed line is included in each panel to mark the 1:1 comparison between experimental and computational data. Error bars represent the standard deviations among experimental replicates.

The *C. bescii* model was validated by comparing model simulations with experimental measurements of biomass and bioproduct yields in the wild-type and engineered strains. These included the wild-type strain DSM 6725 (WT) and the lactate dehydrogenase knockout strain MACB1034 (*Δldh*) (5, 15) (Table 2). The model was first validated based on the prediction of biomass and bioproduct yields when monosaccharides, specifically the glucose (C6) or fructose (C5), were used as sole carbon sources. Both WT and *Δldh* strains were simulated in comparison to experimental measurements (see Data Set S2). A general consistency was observed between the model-predicted and the experimentally measured yields, with the WT model specifically matching the measurements of biomass yields and bioproduct (i.e., lactate, acetate, and pyruvate) productions in batch cultures of *C. bescii* (Fig. 1). Consistent with the experimental data, a slightly higher biomass flux was produced with fructose than with glucose in the *Δldh* model, and higher biomass yields were observed in the WT strain than the *Δldh* strain with either glucose or fructose as the carbon substrate (Fig. 1A). The modeled product yields also showed a high consistency with experimental measurements (Fig. 1B). As expected from experimental studies, the production of lactate was prevented in the *Δldh* model, where the carbon flow was redirected to the production of acetate and pyruvate (see Data Set S2).

**TABLE 2:** Summary of strains modeled in this study^*a*^

Index	Genotype	Parent strain	Strain ID	Reference	Simulation(s)
WT	Wild type		DSM 6725	5	Model validation
*Δldh*	Δ*pyrE* Δ*ldh*	WT	MACB1034	14	Model validation
E1	Δ*pyrE* Δ*ldh adhE^+^ rnf_Na^+^*	Δ*ldh* mutant	MACB1062	10	Model validation
E1M	*adhE^+^ rnf_Na^+^*	WT	This study	This study	Minimal network analysis; basal strain for the testing of engineering designs

a The relevant enzymes are as follows: PyrE, orotate phosphoribosyltransferase; LDH, lactate dehydrogenase; AdhE, bifunctional acetaldehyde/alcohol dehydrogenase; Rnf_Na, sodium ion-pumping reduced ferredoxin NAD oxidoreductase.

Additional validation of the *C. bescii* model was performed by comparing the carbon utilization phenotypes of the WT *C. bescii*, for both model predictions and experimental data (5, 34). A total of 27 carbon compounds were examined, including monosaccharides (C5 or C6), disaccharides, polysaccharides, small organic acids (e.g., acetate and lactate), and polyols (e.g., glycerol, erythritol, and xylitol). The model accurately represented the growth of *C. bescii* on 18 carbohydrates and its lack of growth on 8 other carbon sources, with only one inconsistency in the simulation of dextran utilization (see Fig. S2). While a complete dextran utilization pathway was identified by the *C. bescii* model, existing experimental data showed no growth when dextran was used as a sole carbon source. The simulation of *C. bescii* model for growth on polysaccharides was also validated with crystalline cellulose (Avicel PH-101) as the sole carbon source, using experimental data obtained from the WT strain of *C. bescii* (Fig. 1A). The prediction of biomass yields under a cellulose load of 10 and 50 g/liter agreed well with the experimental data (35). Specifically, a lower biomass yield was observed under a cellulose load of 50 g/liter, likely due to the low percentage of solubilized cellulose and the reduced glucose uptake and utilization under the high cellulose load (see Data Set S2). Overall, the *C. bescii* model was consistent with the experimental data in the prediction of biomass and bioproduct yields.

10.1128/mSystems.01351-20.6 FIG S2 Model validation of *C. bescii* growth/no-growth phenotypes in 27 carbon substrates (5, 34). Download FIG S2, PDF file, 0.04 MB. Copyright © 2021 Zhang et al. 2021 Zhang et al. https://creativecommons.org/licenses/by/4.0/ This content is distributed under the terms of the Creative Commons Attribution 4.0 International license.

### Maximization of ethanol productions in *C. bescii*.

The validated *C. bescii* model was used to identify potential metabolic shifts resulting from the heterogeneous expression of the ethanol production gene *adhE*. To achieve this, random simulations of minimal metabolic networks were performed at three different levels of ethanol production: (i) no-ethanol, where the ethanol production flux was constrained to zero; (ii) half-maximum, where the ethanol production flux was constrained to 50% of the maximum; and (iii) maximum, where the ethanol production flux was constrained to the maximum of model predictions. Model simulations were performed with the E1M strain (Table 2), where AdhE was included to enable the synthesis of ethanol from acetyl coenzyme A (acetyl-CoA), and an Rnf_Na was included based on an existing design of the ethanol-producing E1 strain (10) to evaluate its significance in redox balancing. While the original E1 strain included additional background mutations, such as the deletion of *ldh* and *pyrE*, these background mutations were omitted in the simulation of E1M to allow flexibility in the further engineering of ethanol-producing strains without requiring a specific genetic background.

The minimal metabolism simulations were performed using a modified DSMZ 516 medium with up to 11.8 g/liter cellulose as the sole carbon source, which corresponds to the experimentally measured consumption of cellulose in the E1 strain (10). Up to 1,000 minimal networks were identified for each of the three ethanol production conditions, while maintaining a minimal biomass production that is comparable to the experimentally measured biomass yields (0.41 g of dry weight/liter) under the same medium (see Data Set S3). Examining the occurrence of metabolic reactions among all minimal networks enabled the classification of reactions in the *C. bescii* model into three distinct sets: (i) a core-essential set, representing reactions required in all minimal networks of a simulation condition; (ii) a conditionally essential set, representing reactions required in some but not all minimal networks; and (iii) a nonessential set, representing reactions not used in any minimal networks. Convergence analysis of the minimal networks was performed under the no-ethanol, half-maximum, and maximum-ethanol simulations (see Materials and Methods).

10.1128/mSystems.01351-20.3 DATA SET S3 Model configuration and results summary of the minimal network analysis, which was used for the identification of essential functions for the optimization of ethanol production in *C. bescii*. Download Data Set S3, XLSX file, 0.4 MB. Copyright © 2021 Zhang et al. 2021 Zhang et al. https://creativecommons.org/licenses/by/4.0/ This content is distributed under the terms of the Creative Commons Attribution 4.0 International license.

**FIG 2:**
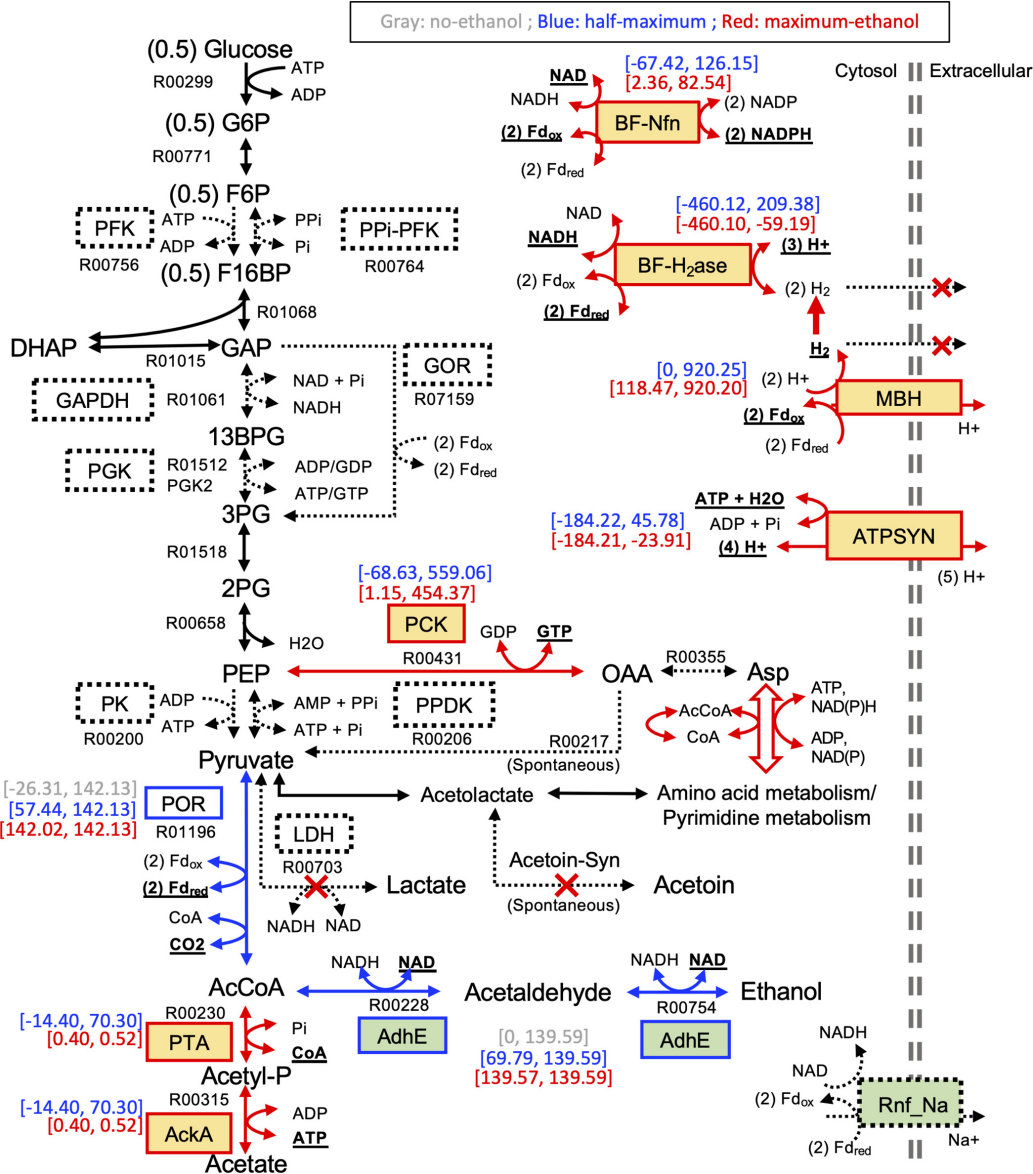
Pathway diagram showing metabolic reactions related to the optimization of ethanol production in *C. bescii*. The solid black arrows indicate essential metabolic reactions in the *C. bescii* model, while the dotted black arrows indicate alternative reactions in the model. The blue arrows indicate reactions that are needed to enable ethanol production. The red arrows indicate reactions that are required for the optimization of ethanol production. The red crosses indicate reactions that should be blocked for the optimization of ethanol production. Functions essential for the optimization of ethanol production are highlighted in yellow background, and engineered functions in the E1 strain of *C. bescii* are on a green background. Metabolic flux ranges are shown for selected functions, with gray representing the flux range under the no-ethanol simulation, blue representing half-maximum ethanol simulation, and red representing the maximum ethanol simulation. Underlined compounds indicate products of metabolic reactions when ethanol production approaches the maximum. Full names of the abbreviated compounds and enzymes can be found in Table S1 in the supplemental material.

Under all three conditions, the classification of core-essential, conditionally essential, and nonessential reaction sets converged before 1,000 rounds of simulations (see Fig. S3). With increasing production of ethanol, a slight increase was observed in the number of core-essential reactions (i.e., from 244 to 275 reactions in the no-ethanol to maximum-ethanol conditions), while a slight decrease was observed in the number of conditionally essential reactions (i.e., from 176 to 124 reactions). Several reactions in the conditionally essential set under no-ethanol simulation were identified in the core-essential set when ethanol production was required. These included AdhE and POR, where POR is required for the production of ethanol due to its essential roles in converting pyruvate to acetyl-CoA, a precursor required for ethanol production via AdhE (see Data Set S3). The utilization of POR also led to the production and release of CO_2_, along with the production of ethanol (Fig. 2).

10.1128/mSystems.01351-20.7 FIG S3 Convergence analysis of 1,000 minimal network simulations in the *C. bescii* model under three distinct constraints of ethanol production: (A) no-ethanol, (B) half-maximum, (C) maximum. Download FIG S3, TIF file, 0.8 MB. Copyright © 2021 Zhang et al. 2021 Zhang et al. https://creativecommons.org/licenses/by/4.0/ This content is distributed under the terms of the Creative Commons Attribution 4.0 International license.

10.1128/mSystems.01351-20.10 TABLE S1 List of abbreviations and full names for the enzymes and metabolites included in Fig. 2. Download Table S1, PDF file, 0.02 MB. Copyright © 2021 Zhang et al. 2021 Zhang et al. https://creativecommons.org/licenses/by/4.0/ This content is distributed under the terms of the Creative Commons Attribution 4.0 International license.

Comparison of minimal networks under half-maximum and the maximum production of ethanol revealed a number of pathways that should be inhibited for the optimization of ethanol production. These included the release of H_2_ and the generation of other products, such as pyruvate, lactate, and acetoin (Fig. 2). In addition, 21 enzymatic functions were classified as core-essential when the ethanol production was constrained to the maximum, while they were conditionally essential under the half-maximum ethanol production (see Data Set S3). These included reactions in ATP/GTP production, amino acids metabolism, and pyrimidine metabolism. The reactions in amino acids and pyrimidine metabolism carried low fluxes and were mainly used for offsetting the overflow of GTP-forming phosphoenolpyruvate carboxykinase (PCK) reaction (Fig. 2). Several ATP-producing reactions, including the F_1_F_0_ ATP synthase (ATPSYN) and the substrate-level phosphorylation pathway composed of phosphotransacetylase (PTA) and acetate kinase (AckA), were also classified as core-essential when ethanol production approaches the maximum. While the ATPSYN was required for the production of ATP, the substrate-level phosphorylation was mainly used for carbon overflow through the trace production of acetate. Finally, the redox reactions, involving MBH, BF-H_2_ase, and BF-Nfn, were also required as core-essential functions for maximizing the production of ethanol. According to model predictions, under maximum ethanol production, MBH carried positive metabolic fluxes that couple the oxidation of Fd_red_ with the production of H_2_. Also, BF-H_2_ase carried negative metabolic fluxes that take the H_2_ produced from MBH into the generation of the reduced cofactors NADH and Fd_red_, while BF-Nfn carries positive fluxes that facilitate the production of NADPH using reducing power from NADH and Fd_red_ (Fig. 2).

### Impact of BF-H_2_ase reversibility and genetic backgrounds on ethanol production.

The minimal network analysis revealed the potential significance of coupling H_2_ production by MBH with the H_2_-consuming direction of BF-H_2_ase in the conservation of reducing compounds for optimizing ethanol production. Such coupling might be feasible, as the BF-H_2_ase has been shown to readily consume H_2_ while reducing NAD and oxidized ferredoxin (Fd_ox_) *in vitro* (29). However, it is not clear whether the MBH and BF-H_2_ase coupling is feasible under physiological conditions. As a counter example, BF-H_2_ase has been determined as a primary source of H_2_ production rather than H_2_ consumption in *C. bescii* (9). This poses the question, does the BF-H_2_ase catalyze a reversible reaction *in vivo*? If not, what is the impact on ethanol production?

**FIG 3:**
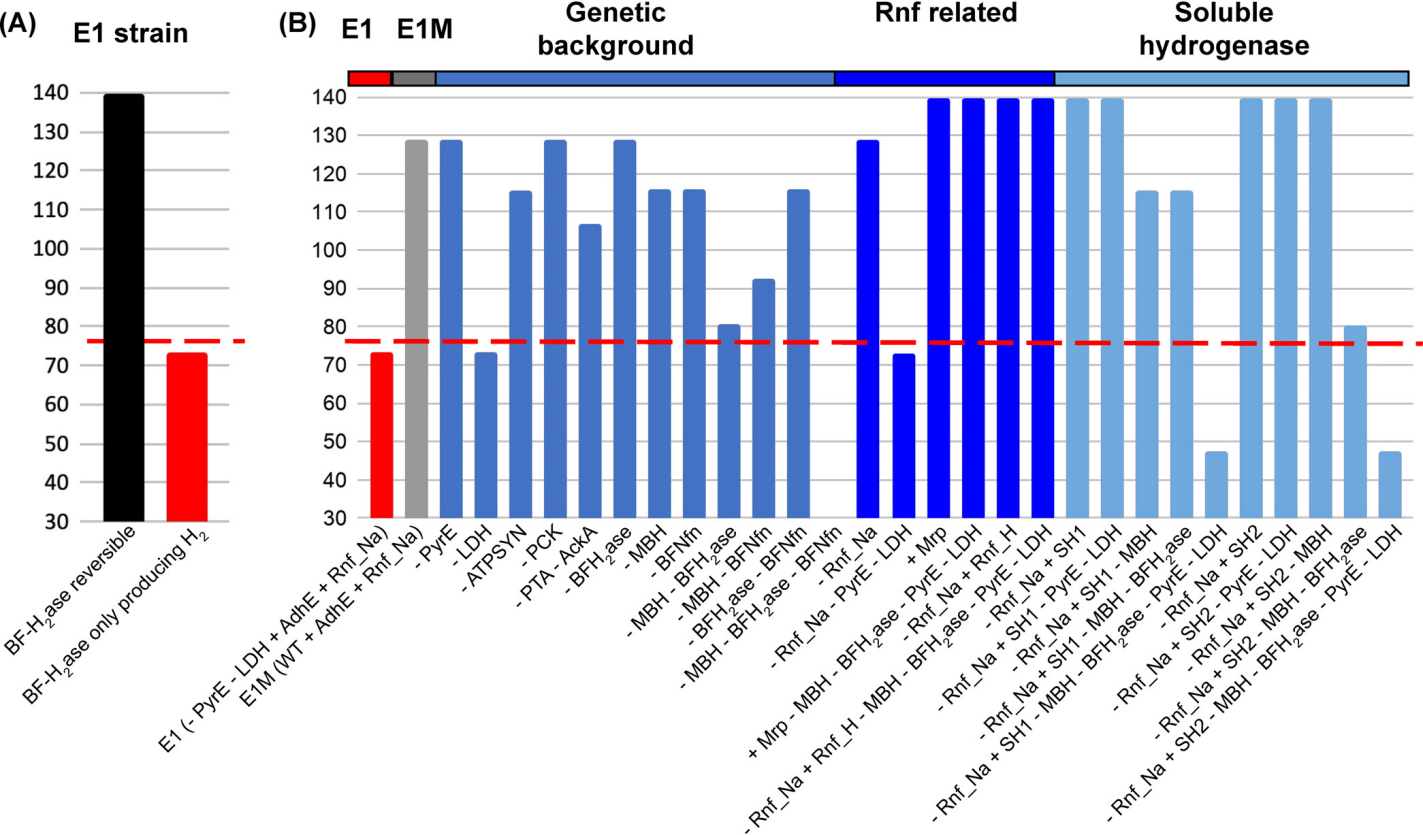
Simulation of maximum ethanol productions for the evaluation of diverse engineering designs. (A) Simulation of ethanol production by the E1 strain with BF-H_2_ase as either reversible (black) or H2-producing only (red). (B) Simulation of engineering designs with the BF-H_2_ase constrained to the H_2_-producing direction. Strain E1M was proposed over the previous engineering design of E1 as a base strain. Further engineering designs were performed with insertion or deletion of genes on the E1M. The symbol “–” indicates the removal of a function, while the symbol “+” indicates the insertion of a function into the E1M model. The red dashed line indicates the experimentally measured ethanol production by the E1 strain.

To address this question, comparisons were performed on the variability of metabolic fluxes between two distinct simulations: one constraining the BF-H_2_ase as a reversible reaction that is capable of carrying flux in both H_2_-producing and H_2_-consuming directions, and another constraining the BF-H_2_ase as an irreversible reaction that only carries flux in the H_2_-producing direction. Simulations were performed on the engineered E1 strain (MACB1062), where ethanol production was optimized in the two BF-H_2_ase settings, followed by the simulation of flux variability for every reaction in the *C. bescii* model (see Data Set S4). When the E1 strain was modeled with a BF-H_2_ase that is H_2_-producing only, it had an optimal ethanol production that was comparable to the experimental measurement (Fig. 3A). This was in contrast to the simulation of E1 when BF-H_2_ase is allowed to consume H_2_, where the predicted ethanol production almost doubled the experimental measurements (Fig. 3A). Therefore, it is likely that the BF-H_2_ase in *C. bescii* could primarily carry out functions in the H_2_-producing direction under physiological conditions.

10.1128/mSystems.01351-20.4 DATA SET S4 Model configuration and results summary of the ethanol optimization analysis, which was used for the evaluation of different engineering strategies. Download Data Set S4, XLSX file, 0.4 MB. Copyright © 2021 Zhang et al. 2021 Zhang et al. https://creativecommons.org/licenses/by/4.0/ This content is distributed under the terms of the Creative Commons Attribution 4.0 International license.

The effect of a H_2_-producing BF-H_2_ase on ethanol production was probed in a diverse range of genetic contexts (Fig. 3B). To better assess the influence of different genetic backgrounds, we used the E1M (Table 2), instead of E1, as the base strain for additional simulations (see Data Set S4). Two genetic backgrounds frequently used for *C. bescii* engineering were considered: one is the deletion of the *pyrE* gene, which is a feature of the stable parent strain for the engineering of ethanol production (15); the other has a deletion of *ldh* gene, which is engineered to block lactate production (14). When the BF-H_2_ase was limited to the H_2_-producing direction, the deletion of *pyrE* had little influence on the ethanol production, while the *ldh* deletion caused a significant reduction of ethanol production (from 129 mM in the E1M base model to 73 mM in the *ldh*-deleted model) (Fig. 3B). In *C. bescii*, the gene that encodes LDH is also responsible for malate dehydrogenase (MDH) activity, which reversibly converts malate to oxaloacetate coupled to the reduction of NAD to NADH (see Data Set S2). Therefore, MDH function is also blocked in the *ldh* deletion mutant. Since MDH is one of the main sources of NADH when the BF-H_2_ase is in the H_2_-producing direction (see Data Set S4), the absence of MDH activity has a negative impact that overrides the benefit of removing LDH for enhancing ethanol production.

Other mutant strains were modeled by inhibiting combinations of the ATP/GTP production functions identified as essential for reaching the maximum production of ethanol, including ATPSYN, PCK, and AckA (Fig. 2). When BF-H_2_ase is constrained to the H_2_-producing direction, a 17% reduction in ethanol yield (from 129 to 107 mM) was observed when the acetate production was blocked, while no influence was observed in the PCK mutant (Fig. 3B). This is likely due to the requirement of an increased flux of carbon overflow in support of substrate-level phosphorylation when the redox balance between Fd_red_ and NADH was shifted with the inhibition of the H_2_-consumption function of BF-H_2_ase (see Data Set S4). In contrast, the deletion of ATPSYN resulted in 10% reduction in the ethanol production when BF-H_2_ase was constrained as H_2_-producing only (Fig. 3B).

Additional mutants were introduced by inhibiting the redox related reactions, BF-H_2_ase, MBH, and BF-Nfn. The deletion of any combinations of the redox functions resulted in a reduction of ethanol yield (Fig. 3B), indicating the significant roles of redox balance in maximizing ethanol production. Among single mutants of these redox functions, the lowest ethanol production was observed with the deletion of MBH. This is likely due to the inhibition of proton pumping by MBH, which limits the ATPSYN-mediated ATP production using proton gradient. Indeed, the predicted ethanol yield of the MBH deletion mutant (116 mM) was comparable to the ATPSYN deletion mutant. In the MBH mutant, the ATPSYN flux was constrained to less than 2% of its minimum flux seen in the E1M base strain, while the flux of substrate-level phosphorylation (i.e., via AckA) was increased up to 50-fold. Both BF-H_2_ase and BF-Nfn were used in the Fd_red_-oxidizing direction in the MBH mutant, mediating redox balance by consuming the Fd_red_ produced by POR (see Data Set S4). Among double mutants of the redox functions, the lowest ethanol production (81 mM) was observed when both MBH and BF-H_2_ase were removed (Fig. 3B). This is likely due to the added effect of reduced proton pumping (e.g., as seen in the single deletion of MBH) and a lack of redox mechanisms that couple the oxidation of Fd_red_ (i.e., produced from POR) to the reduction of NAD; this highlights the significance of both ATP production and redox balance in enabling the maximum ethanol production of *C. bescii*. Further, the deletion of all three redox functions resulted in zero fluxes in both biomass yield and ethanol production, indicating the triple deletion of redox functions would be lethal (see Data Set S4).

### New engineering designs to enhance ethanol production.

As expected based on the above simulations, optimization of ethanol productions in *C. bescii* would require balancing ATP production and redox status and the selection of the appropriate genetic backgrounds for engineering designs. The reversibility of BF-H_2_ase also impacted redox reactions in central carbon metabolism: the NAD-reducing GAPDH function, while forming an alternative pathway to the ferredoxin reducing GOR when the BF-H_2_ase is reversible, was essential when BF-H_2_ase was constrained to only carry flux in H_2_-producing direction (see Fig. S4A and B). This is likely due to the high demand of NADH in the AdhE-dependent ethanol production and lack of a mechanism for coupling Fd_red_ oxidation to the reduction of NAD when BF-H_2_ase functions solely in the H_2_-producing direction (Fig. 2). With the expectation of a primarily H_2_-producing BF-H_2_ase in *C. bescii* under physiological conditions (Fig. 3A), engineering strategies were explored with the H_2_-producing BF-H_2_ase version of the *C. bescii* model to achieve redox balancing and ethanol yield optimization.

10.1128/mSystems.01351-20.8 FIG S4 Variability of metabolic fluxes with ethanol production varying from zero to the predicted maximum at 100 steps. Black (blue) lines with numbers indicate the upper (lower) bounds of fluxes carried out by specific reactions in the designated model. Areas in gray indicate potential flux ranges of the reactions. (A) Simulation of the E1M strain with BF-H_2_ase capable of both consuming and producing H_2_. (B) Simulation of the E1M strain with BF-H_2_ase constrained to the H_2_-producing direction. (C) Simulation following the condition of (B), with the addition of Mrp to the model. (D) Simulation following the condition of (B), with the Rnf_Na replaced by Rnf_H. (E) and (F), Simulation following the condition of (B), with Rnf_Na replaced by the SH1 and SH2 soluble hydrogenases, respectively. Download FIG S4, PDF file, 1.5 MB. Copyright © 2021 Zhang et al. 2021 Zhang et al. https://creativecommons.org/licenses/by/4.0/ This content is distributed under the terms of the Creative Commons Attribution 4.0 International license.

A 77% increase in ethanol production is expected when comparing our original design of the ethanol-producing *C. bescii*, E1 (MACB1062) to a modeled E1M strain (Table 2), based on the observation that the *ldh* deletion inhibited ethanol production with a H_2_-producing BF-H_2_ase (Fig. 3B). Therefore, we chose E1M as the base strain to propose new engineering strategies. An additional feature in the design of the E1 strain included a sodium ion pumping oxidoreductase, Rnf_Na, which was used with the goal of transferring the reducing power of Fd_red_, generated from GOR and POR, to the production of NADH. This initial strategy, however, had limited effect on the ethanol production. As shown in the model simulations, deletion of Rnf_Na from either the E1M base model or the E1 engineered ethanol strain model had minimal influence on the maximum ethanol production (Fig. 3B). Upon closer examination, it was hypothesized that the potential lack of a sodium gradient under physiological conditions may limit the roles of Rnf_Na in redox balancing in *C. bescii* (Fig. 2).

**FIG 4:**
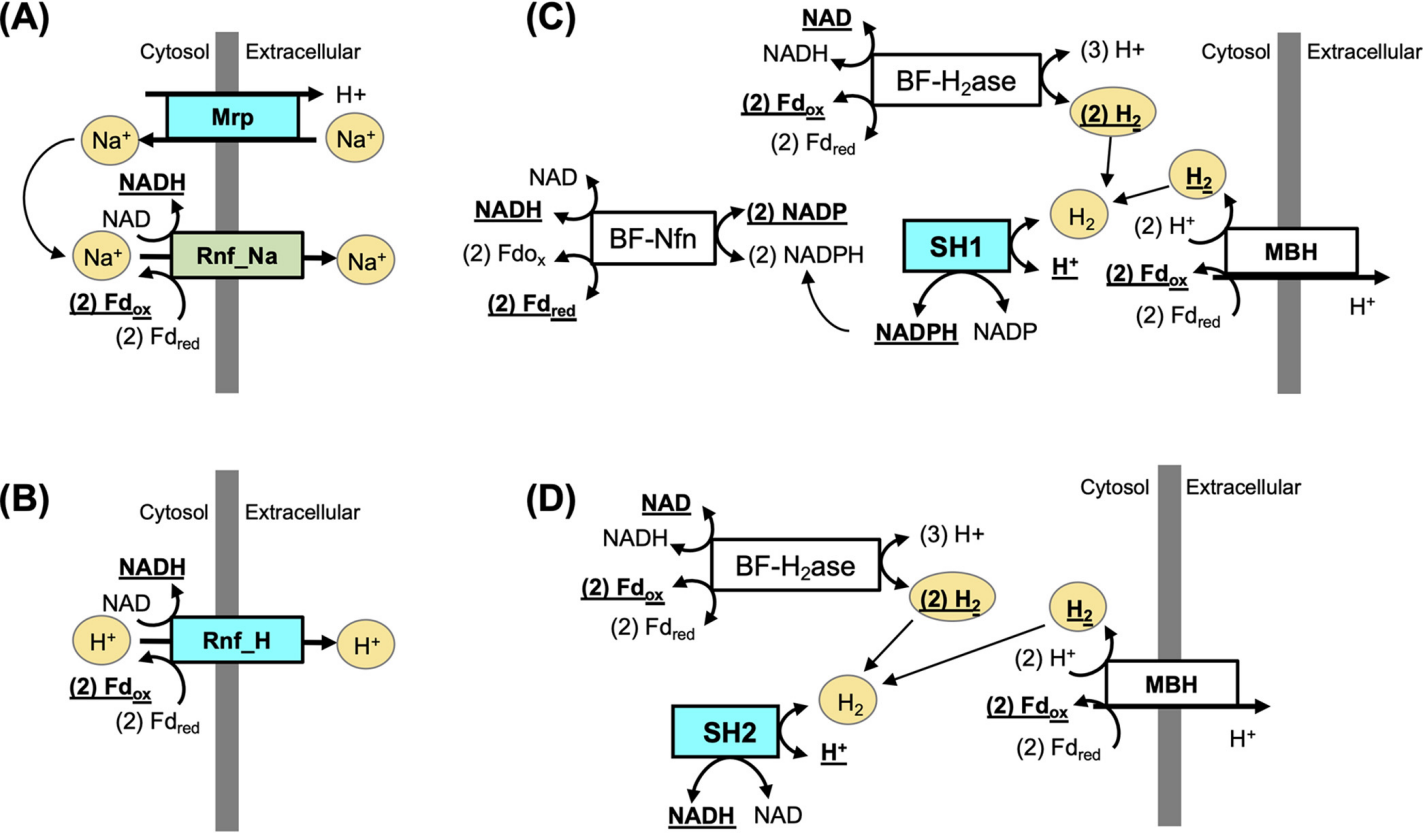
(A to D) Design of four potential engineering strategies predicted by the *C. bescii* model. Boxes with a white background indicate the native metabolic functions in *C. bescii*; boxes with a green background indicate insertion mutants present in existing strains of engineered *C. bescii*; boxes with a cyan background indicate the new engineering strategy proposed in this study. The products of each reaction are represented in boldface with underlines. Key intermediates of each engineering design are represented as ovals with a yellow background.

To test this hypothesis, two new engineering designs were proposed and tested in the *C. bescii* model. In the first design, a membrane-bound sodium-proton antiporter (Mrp) was inserted to the E1M strain to help establish a sodium gradient that can be used to drive the production of NADH by Rnf_Na (Fig. 4A). In the second design, a proton- rather than sodium-driven reduced ferredoxin NAD oxidoreductase (Rnf_H) was introduced to the E1M model in place of Rnf_Na (Fig. 4B). Both engineering strategies were examined under a variation of genetic backgrounds (e.g., deletion of PyrE and LDH) and with the deletion of the H_2_-producing MBH and BF-H_2_ase (Fig. 3B). Optimization of ethanol production in both designs revealed a maximum production of 140 mM (see Data Set S4). The *pyrE* and *ldh* gene deletions were associated with only a minor reduction on the predicted maximum ethanol yield. The deletion of MBH and BF-H_2_ase had no influence on the maximum ethanol production for both engineering designs. Further examination of the flux variability indicated that the introduction of Rnf_Na/Mrp or Rnf_H served as an alternative to the essential proton-pumping function carried out by MBH. Therefore, a zero flux can be achieved for MBH and H_2_ production, even when the BF-H_2_ase was constrained to carry only H_2_-producing fluxes (see Fig. S4C and D).

Besides Rnf functions, additional engineering strategies were explored to couple the H_2_-producing capacity of MBH and BF-H_2_ase with the generation of reduced end products. In a third engineering design, a soluble hydrogenase that is either NADH-producing (SH2) or NADPH-producing (SH1) was introduced in place of the Rnf_Na in E1M. The SH2 and SH1 hydrogenases have been reported to consume H_2_ under physiological conditions in hyperthermophilic archaea (36). Therefore, the introduction of SH2 and SH1 was proposed to transfer the H_2_ production capability (i.e., via MBH and BF-H_2_ase) of *C. bescii* into the production of reducing equivalents for the AdhE mediated ethanol production (Fig. 4C and D). This engineering strategy was proven effective in achieving the maximum ethanol production (Fig. 3). Additional tests were similarly performed on the engineered SH2 and SH1 models against existing genetic backgrounds and redox function deletions. While the deletion of *pyrE* and *ldh* caused only a minor decrease in the maximum ethanol yield, the deletion of MBH and BF-H_2_ase reduced ethanol production by 17% (SH1) and 43% (SH2), and the deletion of MBH and BF-H_2_ase on the genetic background of *pyrE* and *ldh* deletions resulted in a 66% reduction in ethanol yield over the engineered SH2 and SH1 designs (see Data Set S4). Further inspection of the flux variability revealed potential mechanisms leading to the key roles of MBH and BF-H_2_ase in mediating electron flow for ethanol production in the SH1 and SH2 designs. In the SH1 model, MBH is required for the production of proton gradients that drive ATP production via ATPSYN (see Fig. S4E). In the SH2 model, H_2_ production is driven by either MBH or BF-H_2_ase, and BF-Nfn has an essential role in mediating redox balance and facilitating NADPH productions (see Fig. S4F).

## DISCUSSION

The extremely thermophilic, cellulolytic, and hemicellulolytic bacterium, *C. bescii*, is a promising platform to produce desired bioproducts from nonpretreated plant biomass through consolidated bioprocessing. This study provides a first systems-level overview of the *C. bescii* metabolism through a complete GEM reconstruction and used GEM as a tool to inform new engineering designs. The simulation of carbohydrate degradation was enabled through the inclusion of 160 carbohydrate utilization genes (Table 1), including extracellular glycosyl hydrolases, polysaccharide lyases, and ABC superfamily transporters responsible for the uptake of oligo- and monosaccharides (described in detail in Rodionov et al. [33]). The reconstruction of carbohydrate utilization pathways was validated in the *C. bescii* GEM based on extensive validations using biochemical and physiological experimental data (Fig. 1; see also Data Set S2 in the supplemental material). Growth phenotypes were validated for 26 substrates, including C5/C6 monosaccharides, disaccharides, and polysaccharides (see Fig. S2).

The *C. bescii* model was applied in a case study of ethanol production to reveal mechanisms related to ethanol yield optimization. Through a minimal network analysis, several core-essential functions were identified for optimizing ethanol production (Fig. 2). Of significance are POR, which provides acetyl-CoA as a precursor to the AdhE-based ethanol production pathway; ATPSYN, which is responsible for the production of ATP; and three enzymes involved in redox-related functions (i.e., MBH, BF-H_2_ase, and BF-Nfn) that mediate the interconversion of Fd_red_, NADH, and NADPH. Interestingly, the model indicated that the coupling of MBH and the H_2_-consuming direction of BF-H_2_ase is an efficient mechanism for producing NADH, which is required for ethanol synthesis using AdhE. In such a coupling, MBH drives the production of H_2_ while establishing a proton gradient that can be used by ATPSYN for the production of ATP, and BF-H_2_ase utilizes the H_2_ produced by MBH to drive the production of NADH along with a partial recovery of Fd_red_ (Fig. 2).

While the proposed redox coupling strategy between MBH and BF-H_2_ase seems effective, it relies on the BF-H_2_ase functioning in the H_2_-consuming direction. Since BF-H_2_ase has been identified as a primary source of H_2_ production under physiological conditions (9), questions were raised about whether the coupling of MBH and BF-H_2_ase is feasible in engineered strains of *C. bescii*. By simulating the BF-H_2_ase either as reversible reactions or H_2_-producing-only reactions, optimal ethanol production was predicted by the *C. bescii* model under individual or combined perturbations of the ATP/GTP production, redox-related, and genetic background functions (Fig. 3). These analyses further confirmed the significance of redox balance in facilitating maximum ethanol productions in *C. bescii*. More surprisingly, an LDH deletion mutant, which has been used in the design of the E1 strain of *C. bescii*, had limited ethanol production, especially when BF-H_2_ase has a H_2_-producing function. This result corroborates with the experimentally measured ethanol production in the E1 strain, MACB1062 (10), and may serve as an additional indication that BF-H_2_ase primarily produces H_2_ in *C. bescii* (Fig. 3A).

The *C. bescii* model was also used to suggest new engineering strategies for optimizing ethanol yield. Modeling of the existing E1 strain of *C. bescii* showed that the Rnf_Na function, originally introduced to facilitate the Fd_red_-to-NADH conversion (10), was not effective at improving ethanol production due to the lack of a sodium ion gradient (Fig. 3; see also Data Set S4). To circumvent this problem, two new strategies were proposed based on the *C. bescii* model: (i) introduce a membrane bound sodium/proton antiporter, Mrp, to establish the sodium ion gradient required for Rnf_Na function (Fig. 4A), and (ii) replace the sodium-translocating Rnf_Na function with a proton-translocating Rnf_H function (Fig. 4B). According to the modeling results, both engineering strategies have the potential to reach maximum ethanol production of 140 mM using 11.8 g/liter cellulose as the sole carbon source. This almost doubles the experimentally measured yield (76 mM) obtained from existing designs of the AdhE-mediated ethanol-producing strains (Fig. 3B). The designs of Rnf_Na/Mrp and Rnf_H strains are also robust with the existing genetic backgrounds and independent of MBH and BF-H_2_ase coupling, providing alternative solutions in mediating proton pumping and redox balancing. Between the two strategies, the introduction of a Rnf_H might be less useful for *C. bescii* because Rnf_H homologs have been found only in mesophilic organisms with maximum growth temperatures near 40°C (37). In contrast, thermostable versions of Mrp are available, for example, from the hyperthermophilic archaea Pyrococcus furiosus (*T*_max_ = 103°C) and *Thermococcus onnurineus* (*T*_max_ = 90°C) (38, 39). Therefore, the introduction of an Mrp in the existing E1 design could be an optimal solution that would also enable future engineering of the H_2_-producing MBH and BF-H_2_ase functions into *C. bescii*.

Additional engineering strategies were also explored in the *C. bescii* model through the introduction of soluble hydrogenases, SH1 or SH2 (Fig. 3B and Fig. 4C and D). Candidates for the thermostable cytoplasmic SH1 and SH2 hydrogenases are found in the hyperthermophilic archaeal order *Thermococcales* (36, 40, 41). While carrying a similar capability to reach the maximum ethanol production and a similar robustness to existing genetic backgrounds such as in the Rnf_Na/Mrp and Rnf_H designs, the SH1 design is dependent on the proton pumping function of MBH; both SH1 and SH2 designs are dependent on the H_2_-producing functions of MBH or BF-H_2_ase (see Fig. S4E and F).

In summary, a genome-scale metabolic model of *C. bescii* was developed with the goal of optimizing metabolic engineering designs for the production of bio-based chemicals. Using ethanol productions as a case study, several key contributions to ATP production (i.e., ATPSYN), proton pumping (i.e., MBH), and redox processes (i.e., GOR, POR, MBH, BF-H_2_ase, and BF-Nfn) were highlighted that underlie the mechanisms for ethanol yield optimization. The mechanistic understanding acquired from modeling was also applied for the testing of new engineering designs that can be implemented by future experimental studies. The *C. bescii* model, as well as the computational procedures described in this study, will provide a solid foundation for guiding future engineering designs for the production of other desirable bioproducts.

## MATERIALS AND METHODS

### Culture conditions.

*C. bescii* WT and *Δldh* strains were grown under anaerobic conditions with 80:20 (vol/vol) nitrogen-carbon dioxide headspace at 75°C with shaking at 150 rpm. Monosaccharide model validation experiments were conducted in sealed 100 ml serum bottles with 50 ml of modified DSM 516 media, termed DG25 or DF25 (see Data Set S2). Cells for enzymatic activity assays were grown on glucose in triplicate in 2-liter bottles containing 1 liter of medium. Media were prepared as described previously (14) with the following modifications: growth substrates were adjusted from 5 g/liter to 25 mM and NH_4_Cl was increased from 6.16 to 24.11 mM. To quantify the correlation between biomass and protein yields, the *C. bescii* WT strain was grown in a 20-liter custom fermentor. Growth was maintained at 75°C with gas sparging with 80:20 (vol/vol) nitrogen-carbon dioxide at 1.5 liters/h and agitation by dual Rushton stack impellers at 150 rpm. The same modified medium was used with substrate loading (fructose) increased to 100 mM to avoid carbon-limiting growth. Media were prepared at pH 7.2 (room temperature) and allowed to acidify to pH 6.2 (measured at 75°C) during growth, whereupon the pH was maintained at 6.2 by the automated addition of 10% (wt/vol) sodium bicarbonate solution.

### Enzymatic activity assays.

For preparation of whole-cell extracts, 1-liter cultures were grown with 25 mM glucose as described above, to late exponential phase, and harvested by centrifugation at 6,000 × *g* for 10 min (Beckman Avanti J-30I JLA 10.500 rotor). Cell pellets were flash frozen with liquid nitrogen and stored at –80°C. The remaining steps of extract preparation were conducted anaerobically in a Coy chamber. Cell pellets were thawed and resuspended in 25 mM morpholinepropanesulfonic acid (pH 7.0), containing 1 mg/ml lysozyme at a ratio of 3 ml/g (wet weight) cells. Resuspended cells were incubated at room temperature for 10 min, followed by three 10-s rounds of sonication (Qsonica Q55, amplitude 40). For whole-cell extracts, lysates were washed with excess buffer over a regenerated cellulose centrifugation filter (10-kDa Amicon; EMD Millipore) to remove small molecules including redox cofactors. For cytosolic extracts, lysates were clarified by ultracentrifugation at 100,000 × *g* for 1 h (Beckman L90K ultracentrifuge 70.1Ti rotor), and then clarified lysates (S100) were also washed as described above. Both whole-cell and cytosolic extracts were sealed anaerobically and stored at –80°C until use.

Unless otherwise specified, all enzyme assays were performed at 75°C using the S100 fraction in either 3-ml cuvettes (2 ml reaction volume) sealed by silicone stoppers on an Agilent Technologies Cary 100 UV-Vis spectrophotometer with an Agilent Cary series temperature controller or by high-performance liquid chromatography (HPLC; Agilent 1260 Infinity, YMC Hydrosphere C_18_ or Bio-Rad Aminex HPX-87H columns). NAD(P)(H)-dependent activities were measured by changes in absorbance at 340 nm (ɛ = 6.22/mM · cm), and methyl viologen (MV)-dependent activities were measured at 600 nm (ɛ = 13.70/mM · cm).

Pyruvate oxidoreductase (POR), indole-3-pyruvate oxidoreductase (IOR), 2-ketoisovalerate oxidoreductase (VOR), and 2-ketoglutarate oxidoreductase (KGOR) assays were performed as described previously using 1 mM MV, 5 mM pyruvate, 2.5 mM indole-3-pyruvate, 5 mM 2-ketoisovalerate, and 5 mM 2-ketoglutarate substrates, respectively (42). Malate dehydrogenase (MDH) and lactate dehydrogenase (LDH) activities were measured by the NADH-dependent oxidation of oxaloacetate to malate with 1 mM NADH and 2 mM oxaloacetate, as described previously (43). Pyruvate kinase (PK) reactions contained phosphoenolpyruvate (0.5 mM) with 0.5 mM ADP or GDP (no activity observed with GDP). PK assays were linked to pyruvate oxidoreductase by monitoring the pyruvate-dependent reduction of MV (1 mM) and contained 50 mM EPPS buffer (pH 7.0), 5 mM MgCl_2_, 5 mM KCl, 0.1 mM CoA, and 50 μM thiamine pyrophosphate (TPP) in total volume of 2 ml. Pyruvate phosphate dikinase (PPDK) assays mimicked PK assays, with AMP and GMP replacing ADP and GDP (no activity observed with GMP). Phosphoglycerate kinase (PGK) was measured discontinuously in the reverse direction with 3-phosphoglycerate and ATP or GTP, measuring nucleotide hydrolysis by HPLC, with minor modifications to the described protocol (44). Briefly, S100 extract was incubated at 70°C with 2.5 mM MgCl_2_, 2 mM 3-phosphoglycerate, and 100 μM ATP or GTP in 50 mM phosphate (pH 7.0), in a total volume of 1 ml. After incubation, 0.1 ml of the reaction mixture was removed from the reaction vial at 1-min intervals up to 5 min, cooled on ice to halt the reaction, and injected into the HPLC (hydrosphere C_18_ column). Nucleoside monophosphate kinase (NMPK) and nucleoside diphosphate kinase (NDPK) were measured discontinuously by HPLC (hydrosphere C_18_ column) by measuring the appearance and disappearance of ATP, ADP, AMP, GTP, GDP, and GMP after incubation for 5 min at 70°C with cell extracts and MgCl_2_ in 50 mM phosphate buffer (pH 7) (45, 46).

To avoid complications from the native LDH and MDH activities, the following assays were performed using extracts prepared from the *Δldh* strain instead of the WT. Malic enzyme (ME) was assayed in both forward and reverse directions as described previously, with both NADH and NADPH (47). Phosphoenolpyruvate carboxykinase (PCK) was measured in the forward direction with both GDP and ADP by linking oxaloacetate formation with NADH oxidation through the addition of L-MDH from pig heart (Roche, Manheim, Germany), as described previously (48). Activity was determined discontinuously by measuring oxaloacetate formation by HPLC (Aminex HPX-87H column). No ADP linked PCK activity was detected. Phosphoenolpyruvate carboxylase (PEPC) was measured as described previously, in the manner for PCK described, but without the addition of ADP or GDP (49). Oxaloacetate decarboxylase (OAADC) activity was measured in S100 extracts by linking pyruvate formation with NADH oxidation through the addition of L-LDH from rabbit muscle (Roche), as described previously (50). Pyruvate carboxylase (PC) was assayed in S100 similarly to PEPC, by linking oxaloacetate formation from pyruvate to NADH oxidation with pig heart L-MDH (Roche) (51).

### Genome-scale metabolic modeling.

The genome-scale metabolic model of *C. bescii* was reconstructed using the complete genome of strain DSM 6725 (NCBI accession no. NC_012034.1). Manual curations were performed by combining homology searches to public databases, such as KEGG (52), EggNOG (53), UniProt (54), Pfam (55), BioCyc (55, 56), and TCDB (57), a subsystem-based comparative genomics approach for the curation and analysis of carbon utilization pathways (Rodionov et al. [33]), existing literature on the *C. bescii* physiology and metabolic function, and enzymatic assays performed as a part of this study.

The *C. bescii* model was represented in a YAML format following conventions defined by the PSAMM software package (58, 59). All metabolic simulations were performed with PSAMM version 1.0 using the IBM ILOG CPLEX Optimizer version 12.9.0.0 and Python version 3.7. A biomass equation was formulated based on the composition of cell mass in *C. bescii*, including DNA, RNA, protein, lipids, carbohydrates, ions, trace metabolites, lipoteichoic acids, and cell wall components, with the stoichiometry of the overall biomass equation reflecting the gram composition of individual components in 1 g of cell dry weight (see Data Set S1). Individual synthesis equations were used to represent the biosynthesis of macromolecules and the assembly of ion and trace metabolites, with stoichiometry calibrated to represent the millimolar concentrations of basic building blocks in 1 g of each macromolecule.

Metabolic simulations were performed by representing experimental media settings used for the validation of growth and product yield simulations (see Data Set S2). The overall stoichiometry consistency, formula and charge balance of *C. bescii* model were validated by *masscheck*, *formulacheck*, and *chargecheck* implementation in PSAMM (58, 59), where the biomass-related reactions, compound sinks, exchange reactions, and reactions containing compounds with undefined formula (“R” or “X” group presents in formula) were excluded from the formula and charge check and instead manually inspected to ensure the proper formulation. Additional consistency check of the *C. bescii* model was performed with the standardized genome-scale metabolic model testing software, Memote (31), and compared to other published models (Table 1).

### Model validation.

Two strains of *C. bescii* were used for model validations performed in this study: the wild-type strain DSM 6725 (WT) and the lactate dehydrogenase knockout mutant strain MACB1034 (*Δldh*) (5, 15) (Table 2). A linear conversion was applied to convert the experimental measurements of protein yields to the cell dry weight yields (see Fig. S5 in the supplemental material, and the cell density measurements were converted to cell dry weights based on an estimated weight of 8.2 × 10^−10^ mg/cell (60). The WT and *Δldh* strains were simulated to examine the growth and bio-based chemical production using monosaccharides (glucose and fructose) as the sole carbon source, and the WT strain was simulated to examine the growth of *C. bescii* using cellulose (Fig. 1). Simulation of biomass yield was performed with the PSAMM *fba* function for the WT strain and with the *moma2* implementation in the PSAMM *genedelete* function for the *Δldh* strain. Simulation of metabolic products (e.g., lactate, acetate, and pyruvate) were calibrated based on the ratios of their relative concentrations, and the simulation of growth using cellulose was performed by optimizing the biomass yield while calibrating the production of carbon products (e.g., lactate, acetate, and solubilized glucose) based on experimental data (see Data Set S2). Finally, the simulation of growth/no-growth phenotype was performed using the basal medium of DG25 while replacing glucose to a corresponding sole carbon source, and the model predictions were compared to existing growth data of *C. bescii* (5, 34). Detailed simulation settings were provided in Data Set S2.

10.1128/mSystems.01351-20.9 FIG S5 Experimentally identified correlation between protein dry weight and cell dry weight of *C. bescii*. A linearly fitted equation was derived from this analysis and used for the conversion of protein measurements to biomass yields. Download FIG S5, TIF file, 1.1 MB. Copyright © 2021 Zhang et al. 2021 Zhang et al. https://creativecommons.org/licenses/by/4.0/ This content is distributed under the terms of the Creative Commons Attribution 4.0 International license.

### Model-based simulation of metabolic engineering strategies for ethanol production.

A modified DSMZ 516 medium (10, 14) was used for the simulation of engineering strategies for ethanol production, with crystalline cellulose (Avicel PH-101) as the sole carbon source. The minimal network analysis was performed using the PSAMM *randomsparse* function. Three sets of 1,000 *randomsparse* simulations were performed with ethanol production constrained to 0% (no-ethanol), 50% (half-maximum), and 99.99% (maximum) of the predicted maximum. The selection of 99.99% as a threshold for the maximum ethanol simulation was to account for potential uncertainties in solving linear programming models using the CPLEX optimizer. A convergence test was performed for each set of simulations to confirm that the classification of core-essential, conditionally essential, and nonessential reactions were stabilized after 1,000 random simulations (see Fig. S3). A detailed description of model configurations for the minimal network analysis was provided in Data Set S3. The evaluation of different engineering designs was similarly performed by configuring the model with corresponding knock-in/knockout functions while optimizing the production of ethanol (see Data Set S4). Flux ranges under diverse ethanol production thresholds was performed using the PSAMM *fva* function for different engineering designs while setting the BF-H_2_ase as either unconstrained or constrained to the H_2_-producing condition. For each simulation 100 steps were taken by varying the ethanol production constraints from zero to the maximum, while the flux variability was examined for the key metabolic reactions in the *C. bescii* model (see Fig. S4).
