# Supplementary figures and images for: Genome-Scale Metabolic Model of Caldicellulosiruptor bescii Reveals Optimal Metabolic Engineering Strategies for Bio-based Chemical Production
Source: mSystems. 2021 Jun 1;6(3):e01351-20. doi: 10.1128/mSystems.01351-20 (PMC8269263; doi:10.1128/mSystems.01351-20)

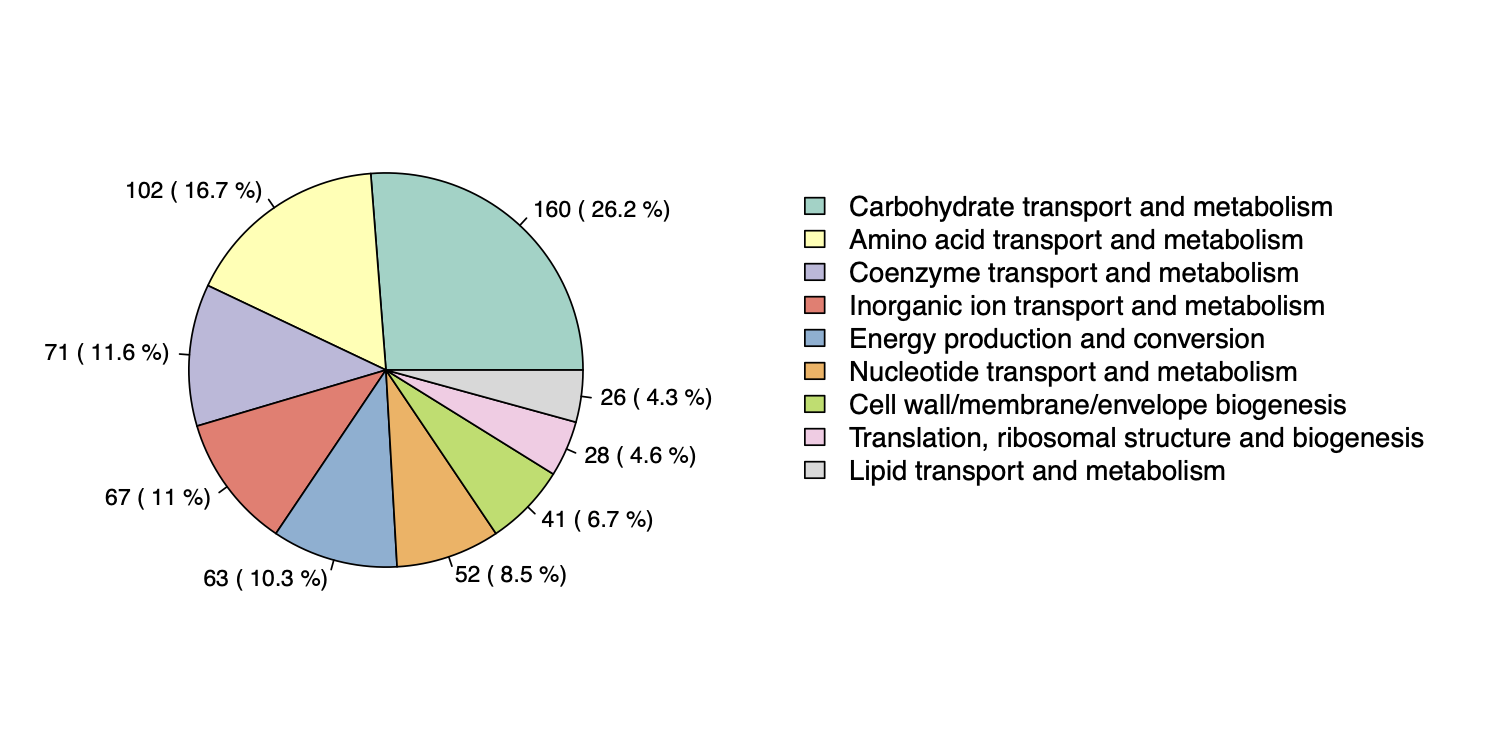

Supplement: FIG S1 [file msystems.01351-20-sf001.tif]

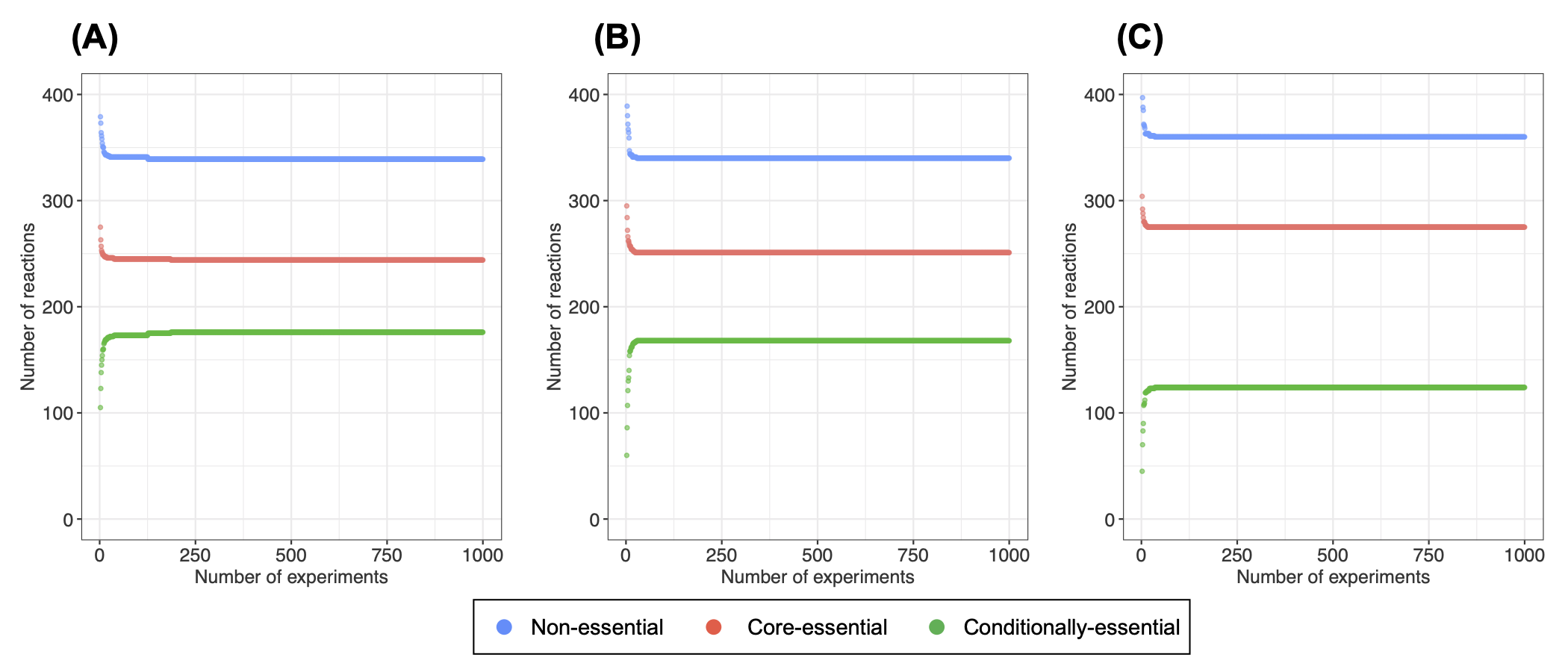

Supplement: FIG S3 [file msystems.01351-20-sf003.tif]

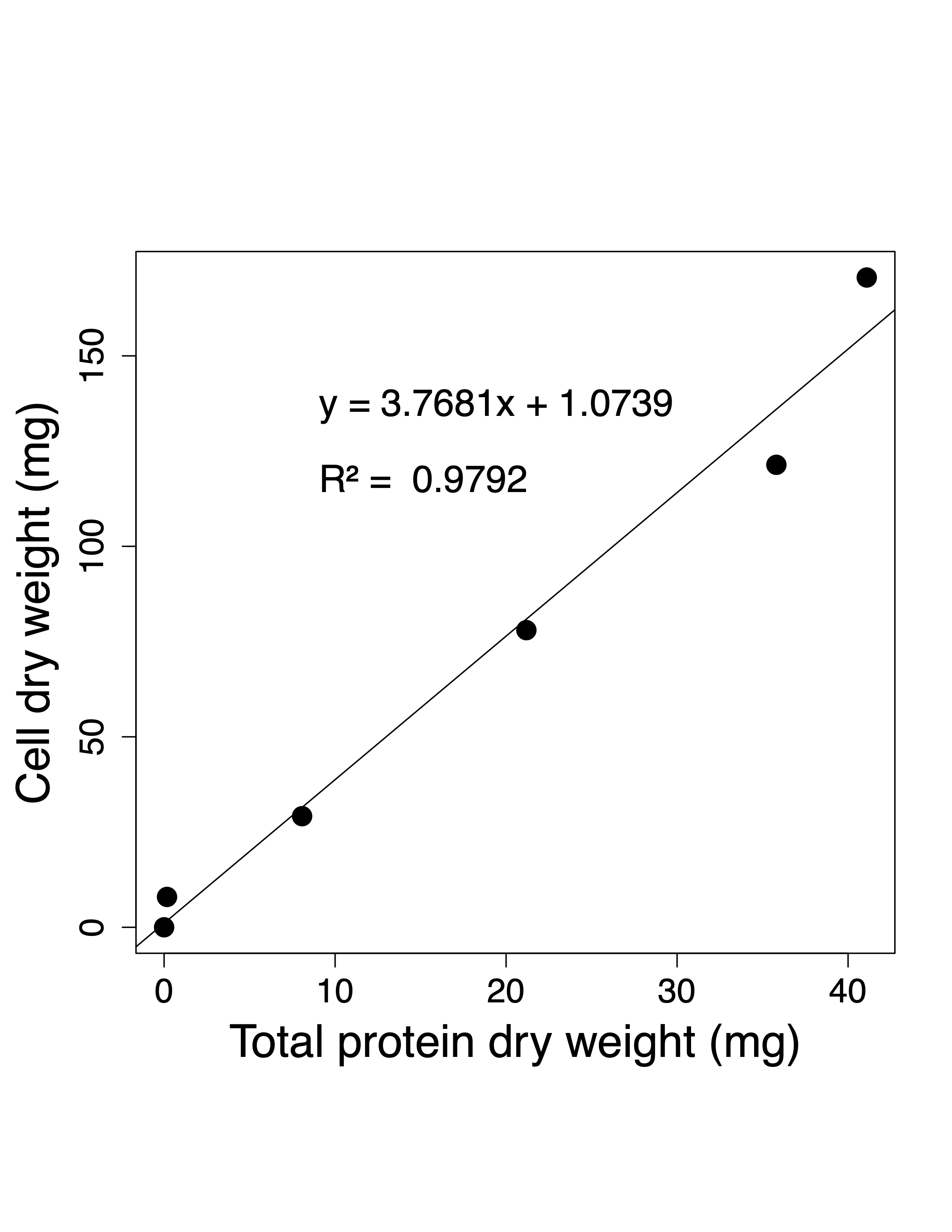

Supplement: FIG S5 [file msystems.01351-20-sf005.tif]
