# Supplementary material for: Genome-Scale Metabolic Model of Caldicellulosiruptor bescii Reveals Optimal Metabolic Engineering Strategies for Bio-based Chemical Production
Source: mSystems. 2021 Jun 1;6(3):e01351-20. doi: 10.1128/mSystems.01351-20 (PMC8269263; doi:10.1128/mSystems.01351-20)
Supplement: FIG S2 [file msystems.01351-20-sf002.pdf]

Substrate	Experimental	Computational	Type
L-arabinose	+	+	Monosaccharide (C5)
xylose	+	+	
glucose	+	+	
fructose	+	+	
galactose	+	+	Monosaccharide (C6)
mannose	+	+	
rhamnose	+	+	
fucose	+	+	
cellobiose	+	+	
lactose	+	+	
maltose	+	+	Disaccharide
melibiose	+	+	
trehalose	+	+	
glycogen	+	+	
starch	+	+	
xylan	+	+	
pectin	+	+	
crystalline cellulose	+	+	Polysaccharide
dextran	-	+	
chitosan	-	-	
inulin	-	-	
mannan	-	-	
acetate	-	-	
lactate	-	-	
glycerol	-	-	Others
erythritol	-	-	
xylitol	-	-	
