# Supplementary material for: Genome-Scale Metabolic Model of Caldicellulosiruptor bescii Reveals Optimal Metabolic Engineering Strategies for Bio-based Chemical Production
Source: mSystems. 2021 Jun 1;6(3):e01351-20. doi: 10.1128/mSystems.01351-20 (PMC8269263; doi:10.1128/mSystems.01351-20)
Supplement: TABLE S1 [file msystems.01351-20-st001.pdf]

Table S1: Abbreviation of enzyme and metabolites names	
**Abbreviation**	**Full name**
**1. Enzymes**	
AdhE	bifunctional acetaldehyde/alcohol dehydrogenase
ldh/LDH	lactate dehydrogenase
AdhA	alcohol dehydrogenase
GOR	glyceraldehyde-3-phosphate (GAP) ferredoxin oxidoreductase
GAPDH	Glyceraldehyde 3-phosphate (GAP) dehydrogenase
POR	pyruvate ferredoxin oxidoreductase
MBH	membrane-bound ferredoxin-dependent [NiFe] hydrogenase
BF-H_2ase	bifurcating [FeFe] hydrogenase
BF-Nfn	bifurcating NADH-dependent ferredoxin NADP+ oxidoreductase I
Rnf_Na	sodium-pumping membrane-bound reduced ferredoxin NAD oxidoreductase
PGK	phosphoglycerate kinase
PCK	GTP-dependent phosphoenolpyruvate carboxykinase
GHs	glycosyl hydrolases
PLs	polysaccharide lyases
pyrE/PyrE	Orotate phosphoribosyltransferase
ATPSYN	F1F0 ATP synthase
PTA	phosphotransacetylase
AckA	acetate kinase
Mrp	membrane-bound sodium-proton antiporter
Rnf_H	proton-driven reduced ferredoxin NAD oxidoreductase
SH1	soluble hydrogenase (NADPH-producing)
SH2	soluble hydrogenase (NADH-producing)
IOR	indole-3-pyruvate oxidoreductase
VOR	2-ketoisovalerate oxidoreductase
KGOR	2-ketoglutarate oxidoreductase
MDH	Malate dehydrogenase
PK	Pyruvate kinase
PPDK	Pyruvate phosphate dikinase
NMPK	Nucleoside monophosphate kinase
NDPK	nucleoside diphosphate kinase
ME	Malic enzyme
PEPC	Phosphoenolpyruvate carboxylase
OAADC	Oxaloacetate decarboxylase
PC	Pyruvate carboxylase
PFK	ATP-dependent 6-phosphofructokinase
PPi-PFK	PPi-dependent 6-phosphofructokinase
**2. Metabolites**	
GAP	glyceraldehyde-3-phosphate
3PG	3-phosphoglycerate
NAD	nicotinamide adenine dinucleotide
Fdred	reduced ferredoxin
Fdox	oxidized ferredoxin
Fd	Ferredoxin
TPP	thiamine pyrophosphate
G6P	D-glucose-6-phosphate
F6P	D-fructose-6-phosphate
F16BP	fructose-1,6-diphosphate
DHAP	dihydroxyacetone phosphate
13BPG	1,3-bisphospho-D-glycerate
2PG	2-phospho-D-glycerate
PEP	phosphoenolpyruvate
OAA	oxaloacetate
Asp	L-aspartate
AcCoA	acetyl-CoA
Acetyl-P	acetyl phosphate
CoA	Coenzyme A
PPi	pyrophosphate
Pi	orthophosphate
