# Supplementary material for: Genome-Scale Metabolic Model of Caldicellulosiruptor bescii Reveals Optimal Metabolic Engineering Strategies for Bio-based Chemical Production
Source: mSystems. 2021 Jun 1;6(3):e01351-20. doi: 10.1128/mSystems.01351-20 (PMC8269263; doi:10.1128/mSystems.01351-20)
Supplement: FIG S4 [file msystems.01351-20-sf004.pdf]

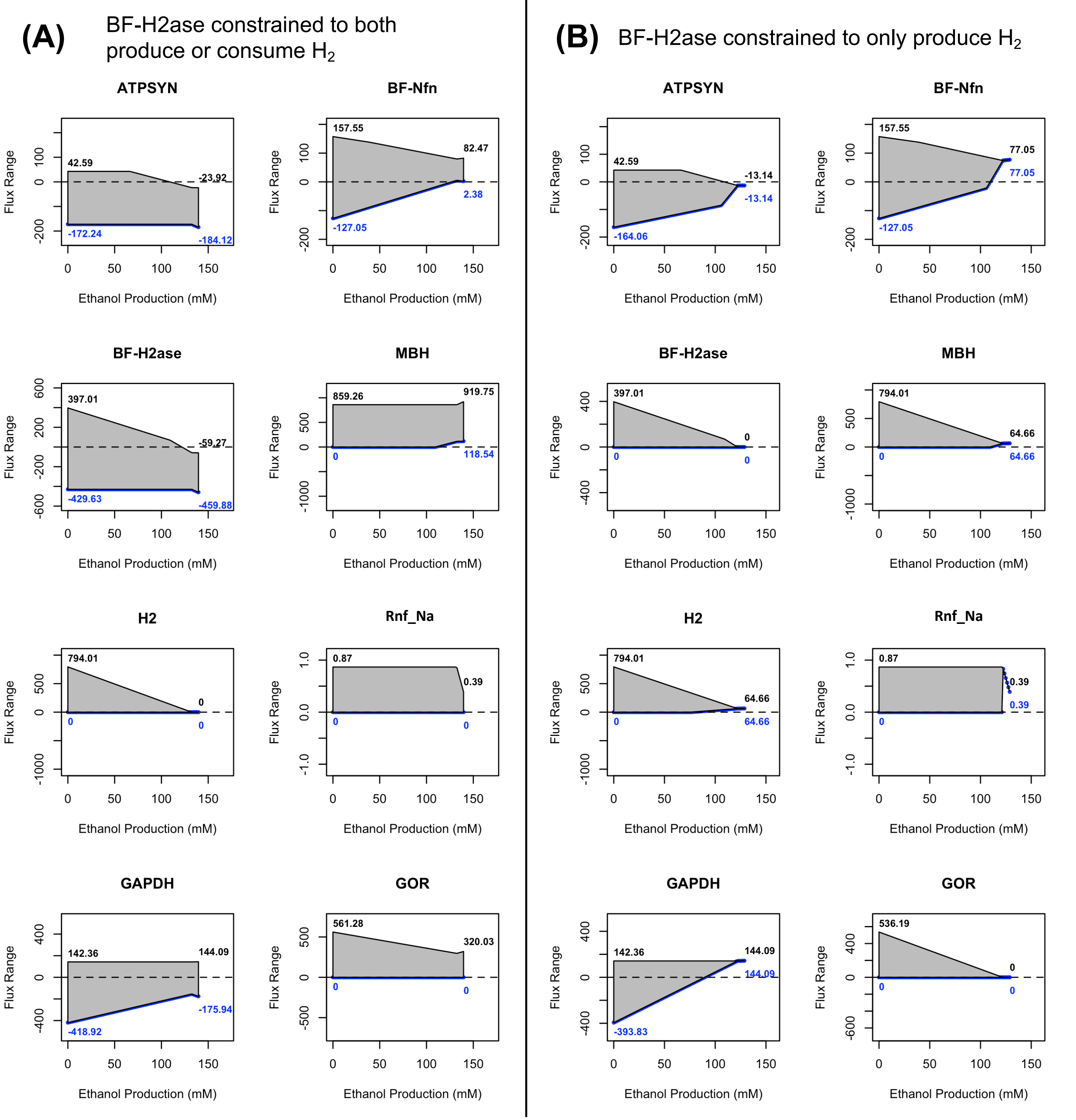

(A)
BF-H2ase constrained to both produce or consume H_2
(B)
BF-H2ase constrained to only produce H_2
ATPSYN
BF-Nfn
BF-H2ase
MBH
H2
Rnf_Na
GAPDH
GOR
Flux Range
Ethanol Production (mM)
42.59
-23.92
-172.24
-184.12
157.55
82.47
2.38
-127.05
397.01
-59.27
-429.63
-459.88
859.26
919.75
118.54
794.01
0.87
0.39
142.36
144.09
-418.92
-175.94
561.28
320.03
-13.14
-164.06
77.05
64.66
-393.83
536.19
0

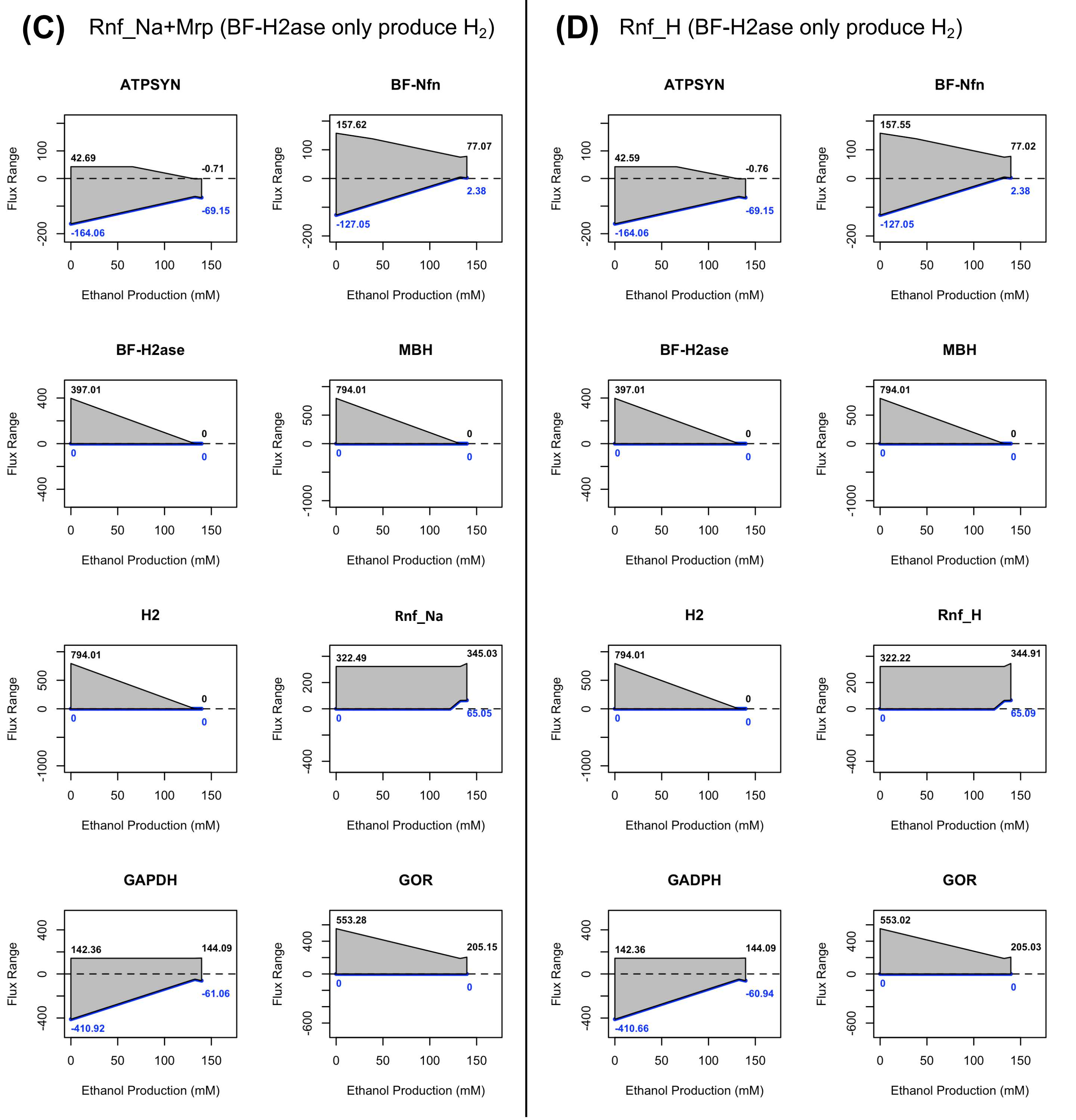
(C) Rnf_Na+Mrp (BF-H2ase only produce H2)
ATPSYN
42.69
-0.71
-164.06
-69.15
BF-Nfn
157.62
77.07
-127.05
2.38
BF-H2ase
397.01
0
0
0
MBH
794.01
0
0
0
H2
794.01
0
0
0
Rnf_Na
322.49
345.03
0
65.05
GAPDH
142.36
144.09
-410.92
-61.06
GOR
553.28
205.15
0
0
(D) Rnf_H (BF-H2ase only produce H2)
ATPSYN
42.59
-0.76
-164.06
-69.15
BF-Nfn
157.55
77.02
-127.05
2.38
BF-H2ase
397.01
0
0
0
MBH
794.01
0
0
0
H2
794.01
0
0
0
Rnf_H
322.22
344.91
0
65.09
GADPH
142.36
144.09
-410.66
-60.94
GOR
553.02
205.03
0
0
Flux Range
Ethanol Production (mM)

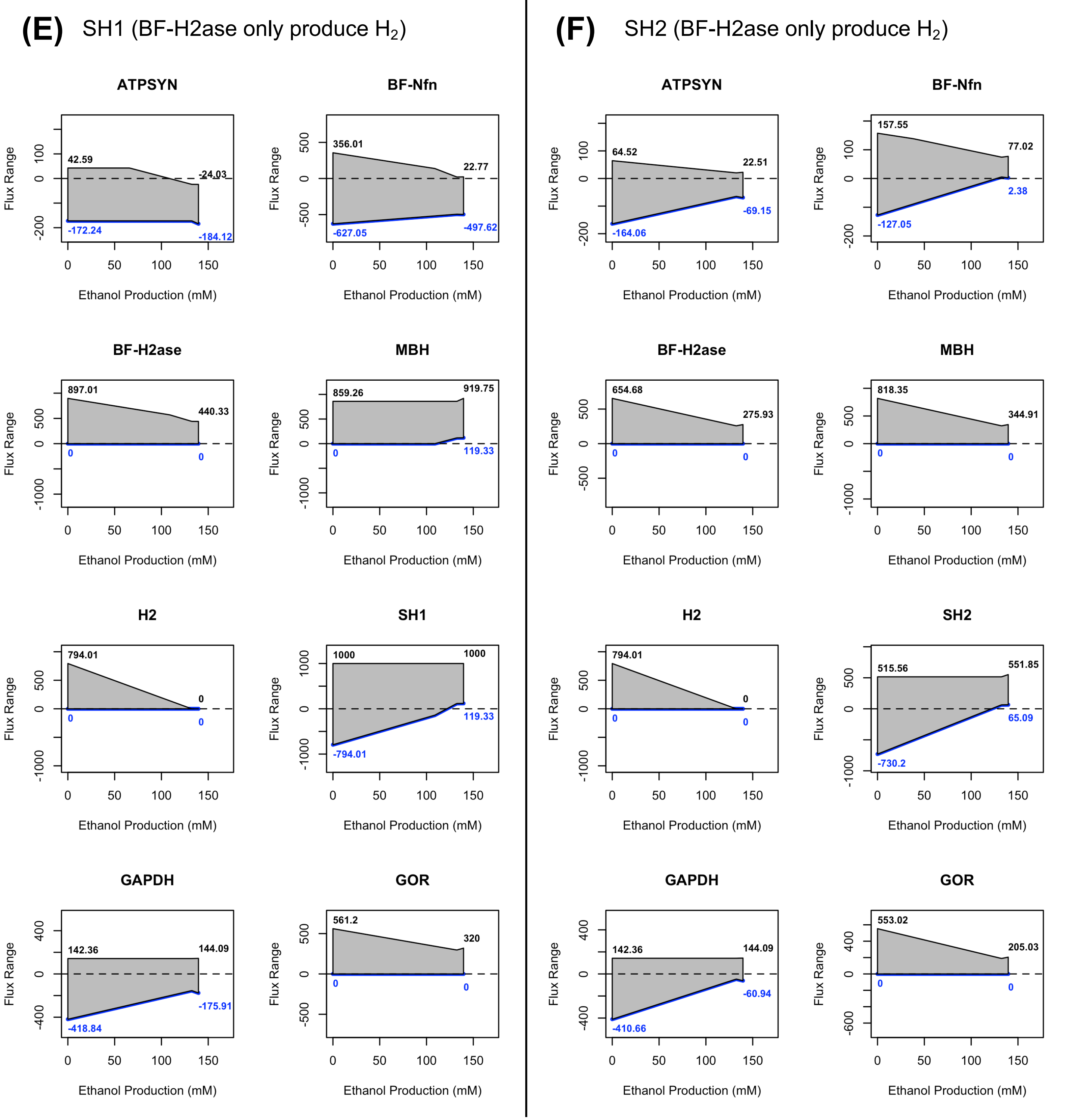
(E)
SH1 (BF-H2ase only produce H_2)
ATPSYN
42.59
-24.03
-172.24
-184.12
BF-Nfn
356.01
22.77
-627.05
-497.62
BF-H2ase
897.01
440.33
0
0
MBH
859.26
919.75
0
119.33
H2
794.01
0
0
0
SH1
1000
1000
-794.01
119.33
GAPDH
142.36
144.09
-418.84
-175.91
GOR
561.2
320
0
0
(F)
SH2 (BF-H2ase only produce H_2)
ATPSYN
64.52
22.51
-164.06
-69.15
BF-Nfn
157.55
77.02
-127.05
2.38
BF-H2ase
654.68
275.93
0
0
MBH
818.35
344.91
0
0
H2
794.01
0
0
0
SH2
515.56
551.85
-730.2
65.09
GAPDH
142.36
144.09
-410.66
-60.94
GOR
553.02
205.03
0
0
Flux Range
Ethanol Production (mM)
